# A new species and new records of *Laelaspis* Berlese (Acari, Laelapidae) from Iran

**DOI:** 10.3897/zookeys.208.3281

**Published:** 2012-07-17

**Authors:** Omid Joharchi, Mahdi Jalaeian, Saeed Paktinat-Saeej, Azadeh Ghafarian

**Affiliations:** 1 Department of Plant Protection, Yazd Branch, Islamic Azad University, Yazd, Iran; 2 Agriculture & Natural Resources Research Center of Khorasan Razavi Province, Plant Protection Department, Mashhad, Iran; 3 Department of Plant Protection, College of Agriculture, Ferdowsi University of Mashhad, Iran; 4 Department of Entomology, Collage of Agriculture, Khorasgan Branch, Islamic Azad University, Isfahan, Iran

**Keywords:** Laelapidae, taxonomy, Formicidae, Iran, myrmecophiles

## Abstract

This paper reports on three species of mites of the genus *Laelaspis* in Iran – *Laelaspis calidus* Berlese from *Pheidole pallidula*, *Laelaspis humeratus* (Berlese) from *Tetramorium caespitum* and *Laelaspis dariusi* Joharchi & Jalaeian, **sp. n.** fromsoil. The new species is described and illustrations provided.

## Introduction

The Laelapidae is one of the largest families of free-living Mesostigmata, but it has not yet achieved a stable classification (Tenorio 1982, Joharchi et al. in press). *Hypoaspis* Canestrini and related genera have had an especially complicated and confusing history, including *Laelaspis* Berlese, 1903, which has often been treated as a subgenus of *Hypoaspis* Canestrini, 1884 (Hunter 1961, Hunter and Glover 1968, Karg 1982, 1993, Faraji et al. 2008). Joharchi et al. (2011) treated *Laelaspis* as a separate genus, and gave a diagnosis and comparison of diagnostic characters for the closely related genera *Gymnolaelaps* and *Pseudoparasitus*. That concept of *Laelapsis* is followed here.

Joharchi et al. previously reported on five species of mites of the genus *Laelaspis* and on several genera associated with ants in Iran (Joharchi et al. in press, Joharchi et al. 2011). Joharchi et al. have previously provided a key to species of *Laelaspis* occurring in the Western Palaearctic Region with a summary of their host associations and biology (Joharchi et al. in press). We now expand the study to include further species in the genus *Laelaspis* Berlese, 1903, mainly associated with ants and soil.

The cosmopolitan genus *Laelaspis* includes 17 species in the Western Palaearctic Region and most species are associated with ants or their nests. However, a few were collected with small mammals or in soil, and most species have only been collected on few occasions, so it is difficult to draw any firm conclusions about their host specificity (Joharchi et al. in press). Six species of *Laelaspis* have been reported previously from Iran (Joharchi et al. in press). Unidentified species of *Laelaspis* were also reported from Iran by Kamali et al. (2001) and Nemati and Babaeian (2010). The purpose of this paper is to describe another species of *Laelaspis* and increase our knowledge of the Iranian fauna of Laelapidae.

## Materials and methods

Mites associated with ants and soil were collected in Alborz, Khorasan, Kerman and Yazd Provinces over a period of two years (2010–2012). Mites were removed from ants’ nests by individual hand picking and by extraction from ant nest and soil material using Tullgren funnels. Mites were cleared in Nesbitt’s solution and mounted in Hoyer’s medium. The nomenclature used for the dorsal idiosomal chaetotaxy is that of Lindquist and Evans (1965), the leg chaetotaxy is that of Evans (1963a) the palp chaetotaxy that of Evans (1963b), and names of other anatomical structures mostly follow Evans and Till (1979). We use the term “lyrifissures” to refer to slit-shaped sensilli, and “pore” for circular or oval-shaped cuticular openings of unspecified function. The holotype and paratypes of the new species are deposited in the Acarological collection, Department of Plant Protection, Yazd Branch, Islamic Azad University (YIAU); paratypes are also deposited in the Jalal Afshar Zoological Museum, College of Agriculture, University of Tehran, Iran (JAZM) and in the Australian National Insect Collection, CSIRO Ecosystem Sciences, Canberra, Australia (ANIC). All measurements in the descriptions are given in micrometres (µm).

## Results

### Genus Laelaspis

Berlese

http://species-id.net/wiki/Laelaspis

Laelaps (Laelaspis) Berlese 1903: 13.

#### Type species.

*Laelaps astronomicus* Koch, 1839, by original designation.

#### Diagnosis.

See Joharchi et al. (2011).

#### Notes on the genus.

*Laelaspis* belongs to a group of genera of Laelapidae in which the genital shield of the female is greatly expanded, so that its posterior margin abuts the anal shield and its lateral margins extend outward behind coxae IV. The expanded genito-ventral shield in these genera captures at least two pairs of ventral setae in addition to the genital setae on the the extreme edges of the shield. *Laelaspis* is distinguished from *Gymnolaelaps* by its two-tined palp tarsal claw, the absence of pre-sternal shields, and the presence of two distinct Λ-shaped lines on the genital shield. *Laelaspis* differs from *Pseudoparasitus* because *Pseudoparasitus* has at least two pairs of setae on the surface of the genital shield, well separated from the edges of the shield, while all the genital setae of *Laelaspis* and *Gymnolaelaps* are on the extreme edges of the shield.

### Laelaspis calidus

Berlese

http://species-id.net/wiki/Laelaspis_calidus

Laelaspis calidus Berlese 1924: 255; Hunter 1961: 676.
Hypoaspis (Laelaspis) calidus .– Aswegen and Loots 1970: 27.
Hypoaspis (Laelaspis) calida .– Karg 1982: 250; 1989: 120.

#### Specimens examined.

Six females, Anar, Kerman, 53°30'N, 18°55'E, alt. 1152 m 10 November 2011, O. Joharchi coll., in nest of *Pheidole pallidula*.

#### Notes.

*Laelaspis calidus* was described from east Africa (Berlese 1924), also has been recorded at Kilimanjaro near Marangu from moss and litter (Aswegen and Loots 1970) and has not been reported since. It is easily recognised by the bidentate movable digit and the seven-toothed fixed digit, the serrated postanal seta and seta Z5 two to three times as long as J5. This species has been found from moss and litter, but has not been reported from the nests of ants. It is now recorded in Iran for the first time from the ant nests.

### Laelaspis dariusi

Joharchi & Jalaeian sp. n.

urn:lsid:zoobank.org:act:BB2DA9B6-4ACF-4F3B-A516-3E33EA3AD1F0

http://species-id.net/wiki/Laelaspis_dariusi

Figures 1–8

#### Specimens examined.

Holotype, female, Iran, Khorasan Razavi Province, Kalate Naderi (Laeen), 37°07'N, 59°29'E, alt. 858 m, 26 Mar 2010, S. Paktinat-saeej coll., in soil of apple orchard. Paratypes, seven females, same data as holotype (in YIAU, JAZM and ANIC).

#### Description of the female.

**Figures 1–8.**
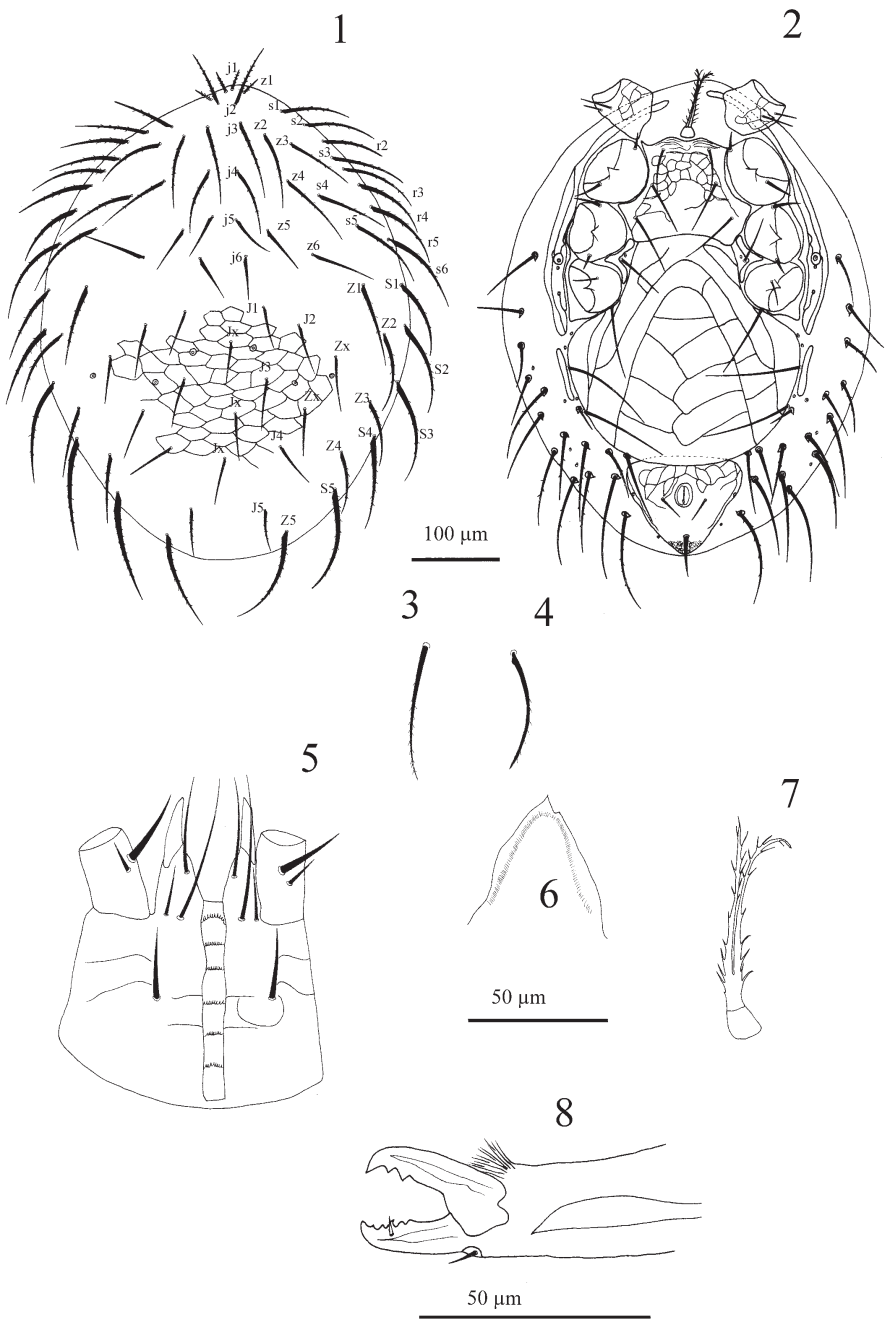
*Laelaspis dariusi* Joharchi and Jalaeian sp. n., female. **1** Dorsal shield **2** Ventral idiosoma **3** Seta Z5 enlarged **4** Dorsal shield seta s3 **5** Hypostome **6** Epistome **7** Tritosternum **8** Chelicera.

Figures 1–8. *Dorsal idiosoma*. Dorsal shield length 524–534, width 406–426 (n = 8) (Fig. 1). Shield oval shaped, with reticulation, more distinct in opisthonotal region; with 39 pairs of long setae, 22 podonotal, 17 opisthonotal, including two pairs of Zx setae between J and Z setae, almost all setae slightly swollen at base, with pointed tip (Fig. 4), podonotal setae very long, reaching well past base of next posterior setae, setae of central area of dorsal shield decreasing in length from anterior to posterior (j3, z2 74-82, j4 69-74, j6, J1, J3 54-57), lateral setae thicker than central setae, almost all marginal setae including Z5 slightly serrated (Fig. 3), length 89–99, almost double length of J5, length 45–50; opisthonotal region with three unpaired supernumerary seta Jx in each specimen. Shield with three pairs of large circular to oval-shaped pores, other pores inconspicuous.

*Ventral idiosoma* (Fig. 2). Tritosternum with columnar base (15–17 long × 9–10 wide), paired pilose laciniae, length 67–69 (Fig. 7), pre-sternal shields absent, pre-sternal area with some weak transverse lines. Sternal shield length 111–116, narrowest between coxae II (87–89) widest between coxae II & III (151–153), with slightly concave posterior margin and undulating anterior margin, with three pairs of long and smooth sternal setae, *st*142–47, *st*2 59-62, *st*3 67-69, reaching well past base of next posterior setae, one pair of lyrifissures adjacent to setae *st*1, a pair of larger lyrifissures between *st*2 and *st*3; antero-lateral surface of sternal shield with lineate ornamentation, central area smooth. Metasternal platelets absent, metasternal setae *st*4 (27–32) and metasternal pores located in soft skin; endopodal plates II/III fused to sternal shield, endopodal plates III/IV elongate, narrow, curved. Genital shield broad, length 277–285, maximum width 248–260, posterior margin rounded, abutting anal shield, surface with characteristic ornamentation including distinct Λ-shaped lines and polygonal ornamentation, bearing the long genital setae *st*5 (87–89) and two pairs of long setae (89–99) on its lateral edges. Paragenital pores located on soft skin lateral to shield behind coxae IV. Anal shield subtriangular, length 104–109, width 126–131; its anterior half with lineate ornamentation and a pair of lateral pores; post-anal seta 42–45 µm, longer and thicker than para-anal setae, 22–25. Opisthogastric skin with long, narrow and oval metapodal plates (62–64 long × 8–10 wide) very close to genital shield, and 15 pairs of slightly serrate setae, each arising on small sclerotised platelet, and seven pairs of pores. Exopodal plates forming subtriangular extensions behind coxae IV, narrow elongate exopodal plates II/III not fused to peritrematal shield. Peritreme extending from coxa IV to anterior of coxa I, peritrematal shield narrow, post-stigmatal section conspicuous, with two pairs of pores.

*Gnathosoma*. Epistome triangular, smooth (Fig. 6). Hypostomal groove with six rows of denticles each bearing 8–10 small teeth, and smooth anterior transverse line. Hypostome with four pairs of setae, internal posterior hypostomal setae h3 longest (Fig. 5). Corniculi robust and horn-like, reaching mid-level of palp femur. Palp chaetotaxy: trochanter 2 (v1 thick), femur 5, genu 6, tibia 12, tarsus 15; all setae smooth and needle-like, palp tarsal claw two-tined. Fixed digit of chelicera with six blunt teeth (Fig. 8); pilus dentilis short and robust; dorsal seta short, prostrate; movable digit with two teeth; arthrodial membrane with a rounded flap and short filaments.

*Legs*. Legs II and III short (302-312, 282-288), I and IV longer (430-446, 372-392). Leg I: coxa 0 0/1 0/1 0, trochanter 1 1/1 0/2 1 (*ad* thick), femur 2 3/2 2/2 2 (*ad2*, *ad3*, *al1*, *pl1* and *pl2* thick), genu 2 3/2 3/1 2 (all dorsal thick), tibia 2 3/2 3/1 2. Leg II: coxa 0 0/1 0/1 0 (all setae thick), trochanter 1 0/1 1/2 1, femur 2 3/1 2/2 1 (*ad1*, *pd2* and *pv1* thick), genu 2 3/1 2/1 2 (all ventral thick), tibia 2 2/1 2/1 2 (all ventral thick). Leg III: coxa 0 0/1 0/1 0 (all setae thick), trochanter 1 0/1 0/2 1 (*al* and *av* thick), femur 1 2/1 1/0 1 (*ad1* and *ad2* thick), genu 2 2/1 2/1 1 (ventral setae thick), tibia: 2 1/1 2/1 1(ventral setae thick). Leg IV: coxa 0 0/1 0/0 0, trochanter 1 0/1 0/2 1 (*av* thick), femur 1 2/1 1/0 1 (*al* long, *ad*1 and *ad*2 thick), genu 2 2/1 3/0 1 (ventral thick), tibia 2 1/1 3/1 2; all setae fine and needle-like unless otherwise noted. Tarsi I-IV with 18 setae 3 3/2 3/2 3 + *mv*, *md*. All pre-tarsi with a pair of claws and a long thin membranous ambulacrum.

*Insemination structures* not seen, apparently unsclerotised.

#### Etymology.

The species is named in memory of Darius I (Old Persian: *Dārayava(h)uš*), also known as Darius the Great, was the third king of the Achaemenid Empire, who proved to be a strong and wise ruler and he was tolerant toward other religions and cultures, promoted learning, agriculture, forestation, and the construction of highways. He also built the great palace cities of Susa and Persepolis.

#### Notes.

*Laelaspis dariusi* differs from all other species in the genus by its dorsal shield setae in central area decreasing in length from anterior to posterior, seta Z5 much longer than J5; seta v1 on the palp trochanter thick, sternal setae long and smooth, long enough to well past base of next posterior setae, movable digit of chelicera with two teeth and fixed digit of chelicera with six blunt teeth.

### Laelaspis humeratus

(Berlese)

http://species-id.net/wiki/Laelaspis_humeratus

Laelaps (Laelaspis) humeratus Berlese 1904: 425.
Hypoaspis humerata .— Evans and Till 1966: 212; Lapina 1976: 43.
Laelaspis humeratus .— Hull 1925: 210; Willmann 1951: 113; Hunter 1961: 675; Salmane 2001a: 131; Salmane 2001b: 34; Salmane and Brumelis 2010: 390.
Hypoaspis (Laelaspis) humerata .— Karg 1979: 102; 1982: 250; 1989: 120.
Hypoaspis humerata .— Bregetova 1977: 545.

#### Specimens examined.

One female, Alborz, Karaj, 35°56'N, 51°22'E, alt. 2000 m, 11 July 2011, O. Joharchi coll., in nest of *Tetramorium caespitum*.

#### Notes.

*Laelaspis humeratus* was described from Luxemburg (Berlese 1904), and has been recorded from Latvia (Lapina 1976; Salmane 2001a, 2001b), Russia and Austria (Bregetova 1977), and England (Hull 1925; Evans and Till 1966). This species was found associated with at least two genera of ants (*Lasius* and *Tetramorium*), free-living in soil, litter and meadows, and from the nests of mammals. This species is easily recognised by the large number of long, thick and wavy opisthonotal setae, the bidentate movable digit and the tridentate fixed digit. Haddad Irani-Nejad et al. (2003) recorded an unidentified species as *Laelaspis* near *humerata* (Berlese 1904), but the identity of that species cannot be confirmed because the specimens have been lost, so this is the first record of *Laelaspis humeratus* from Iran.

## Discussion

Before the start of this study, six species of *Laelaspis* had been reported from Iran. We have added new information on *Laelaspis calidus* and *Laelaspis humeratus*.

Joharchi et al. have previously discussed the distinction between *Laelaspis* and *Gymnolaelaps* and *Pseudoparasitus* (Joharchi et al. 2011). The biology of most species of *Laelaspis* has not been studied, but the limited information that is available shows that they are predatory (Hunter 1964). *Laelaspis* appears to be a genus of predators that feed on other small invertebrates in their hosts’ nests, but are not harmful to the ants. High populations of acarids may be harmful to ants, so the presence of predators such as *Laelaspis* may be beneficial, forming a symbiotic relationship with its ant hosts. The ecological role of *Laelaspis* in mammal nests is also unknown, but it appears likely that they are predators, feeding on other nest inhabitants such as acarid mites (Rasmy et al. 1987).

## Supplementary Material

XML Treatment for Laelaspis

XML Treatment for Laelaspis calidus

XML Treatment for Laelaspis dariusi

XML Treatment for Laelaspis humeratus
